# Morgan County Medical Society

**Published:** 1867-03

**Authors:** C. T. Wilbur

**Affiliations:** Secretary


### PROCEEDINGS OF MEDICAL SOCIETIES.

### MORGAN COUNTY MEDICAL SOCIETY.

The Society met at the Court House in Jacksonville, at 2 o'clock P.M., on Thursday the 14th day of February.

In the absence of the President and Vice-President Dr. Askew was appointed President *pro tem.*

The minutes of the last meeting were read and approved as printed in the JOURNAL. Drs. DeLeuw and C. A. Edgar were nominated for membership.

As no one of the individuals regularly appointed for the exercises of the meeting were prepared with papers, Dr. Malone was requested to read a paper on the Calabar Bean.

After giving its natural history, he proceeded to its action upon animals and upon the human system—when administered in the usual way—by subcutaneous injection and in other ways. Its local influence upon the eye was more particularly commented upon, and the experiments of quite a number of foreign scientific men were mentioned, and the results of those experiments were, briefly, that the local action of this drug produced first, a sensation of "strong tension in the neighborhood of the ciliary body," and second, "astigmatism or a state of irregular refraction in the eye in which the rays are not brought to one focus, but converge at different distances so as to form two linear images at right angles to each other." This peculiar condition of vision was remedied by concave glasses of proper foci. This astigmatic state existed eighteen hours after its application. Numerous experiments have proven that it invariably causes prompt contraction of the pupil, and that the contraction reaches its maximum in the course of an hour.

Dr. Malone was requested by the Society to publish his paper in some medical journal.

Dr. Prince had prepared and read "the chief points in a case of forward dislocation of the tibia on the astragalus with fracture of fibula, in which at the end of nearly three months the dislocation was found still to exist, having either never been reduced or having become re-dislocated. The displacement at the end of this period was reduced and the parts kept in position by an outside splint of wood, pasteboard, and bandage. The result was a prosecution for damages against the physician first in attendance, on account of alleged malpractice (tried three years after the receipt of the injury), the jury finding for the defendant.

The doctor read the instructions given by the judge to the jury in this case.

Dr. Edgar remarked that he was much interested in the case. The suit evidently grew out of malice. The man was hunted down by his medical brethren of the same county. It arose from want of professional courtesy.

The doctor hoped that medical men would rise above their petty feelings of jealousy and rivalry, and cease to give cause to the members of other professions to hold them up as proverbially quarrelsome.

Dr. Edgar, having prepared some notes on the early history of the introduction of mercurials into the materia medica, was requested to read them to the Society, which he accordingly did. They were listened to with marked attention and interest by the Society.

Dr. Reed made some remarks upon the use of mercurials.

The Society then adjourned to meet on the second Thursday in March, at half-past 1 o'clock P.M., at the Court House, in Jacksonville.

C. T. WILBUR, M.D., *Secretary*.

